# Omega-3 fatty acids for breast cancer prevention and survivorship

**DOI:** 10.1186/s13058-015-0571-6

**Published:** 2015-05-04

**Authors:** Carol J Fabian, Bruce F Kimler, Stephen D Hursting

**Affiliations:** Department of Internal Medicine, University of Kansas Medical Center, 3901 Rainbow Boulevard, Kansas City, KS 66160 USA; Department of Radiation Oncology, University of Kansas Medical Center, 3901 Rainbow Boulevard, Kansas City, KS 66160 USA; Department of Nutrition, University of North Carolina, 135 Dauer Drive, Chapel Hill, NC 27599 USA

## Abstract

Women with evidence of high intake ratios of the marine omega-3 fatty acids eicosapentaenoic acid (EPA) and docosahexaenoic acid (DHA) relative to the omega-6 arachidonic acid have been found to have a reduced risk of breast cancer compared with those with low ratios in some but not all case–control and cohort studies. If increasing EPA and DHA relative to arachidonic acid is effective in reducing breast cancer risk, likely mechanisms include reduction in proinflammatory lipid derivatives, inhibition of nuclear factor-κB-induced cytokine production, and decreased growth factor receptor signaling as a result of alteration in membrane lipid rafts. Primary prevention trials with either risk biomarkers or cancer incidence as endpoints are underway but final results of these trials are currently unavailable. EPA and DHA supplementation is also being explored in an effort to help prevent or alleviate common problems after a breast cancer diagnosis, including cardiac and cognitive dysfunction and chemotherapy-induced peripheral neuropathy. The insulin-sensitizing and anabolic properties of EPA and DHA also suggest supplementation studies to determine whether these omega-3 fatty acids might reduce chemotherapy-associated loss of muscle mass and weight gain. We will briefly review relevant omega-3 fatty acid metabolism, and early investigations in breast cancer prevention and survivorship.

## Introduction

Although the predominant driving force in breast carcinogenesis has been thought to be hormonal, cytokine production and inflammation are also being recognized as important in breast cancer development and progression [1,2]. A progressive increase in activated macrophages and T cells is observed between normal breast tissue, proliferative breast disease, and breast cancer [3,4]. The stimulus for the increase in inflammatory cell infiltration observed with proliferative breast disease and breast cancer is unknown but probably has varying etiologies including immunogenic gene alterations in epithelial cells [5], reaction to breakdown of basement membrane components [4], and for obese women excess cytokine production from dysfunctional adipocytes [6].

The long-chain omega-3 fatty acids eicosapentaenoic acid (EPA) and docosahexaenoic acid (DHA) are important in generating bioactive lipid mediators important in inflammation resolution [7]. As key components of phospholipid membranes and lipid rafts that serve to organize or separate molecules, these fatty acids also affect cell signaling thought to impact breast carcinogenesis [8-12]. The ability of long-chain omega-6 fatty acids to modulate inflammation and other physiologic processes is dependent on concomitant levels of the proinflammatory omega-6 arachidonic acid (AA) as well as an individual’s genetic makeup governing lipid metabolism [13-16].

Interest in the use of supplementary omega-3 fatty acids to reduce risk of cancer and other chronic debilitating conditions, including cardiovascular disease and cognitive impairment, stems from several longstanding avenues of investigation: 1) an increased incidence of breast cancer and heart disease in western societies with low omega-3:omega-6 fatty acid intake ratios; 2) a very low incidence of these two conditions in populations with high marine omega-3 fatty acid intake (Japan and natives of Alaska and Greenland); 3) a dramatic increase in the incidence of breast cancer and cardiovascular disease in cohorts from low-incidence populations who migrate to western countries and/or adopt a western diet [15,17]; and 4) the demonstrated importance of adequate DHA in retinal and brain development and cognitive function [18,19].

Although the ideal total omega 3:omega-6 intake ratio has not been defined, a ratio approaching 1:1 or 1:2 similar to that of precivilized man is generally accepted as associated with a low incidence of diseases characterized by chronic inflammation, and therefore is desirable [16,20]. By the early 1900s the omega 3:omega-6 intake ratio in the United States was estimated at 1:5, probably due to the high dietary content of corn oil products and corn-fed animals. Today, largely due to the >1,000-fold increase in use of soybean oil in the last several decades, the dietary omega 3:omega-6 intake ratio is now 1:10 or lower [16,21]. Although much of the imbalance is probably due to the increase in omega-6 consumption, it has been suggested that the most practical remedy may actually be to increase long-chain or marine omega-3 intake rather than to attempt to markedly reduce omega-6 intake [22,23].

We will briefly review omega-3 and omega-6 fatty acid metabolism and function, preclinical mechanistic and prevention studies, as well as selected case–control and prospective cohort studies, and ongoing trials relevant to breast cancer prevention. Reports dealing with omega-3 fatty acids and breast cancer recurrence as well as other relevant survivorship topics including insulin resistance and obesity, cardiovascular disease and cognition will also be discussed.

## What are omega-3 and omega-6 fatty acids and how do they work?

**Figure 1:**
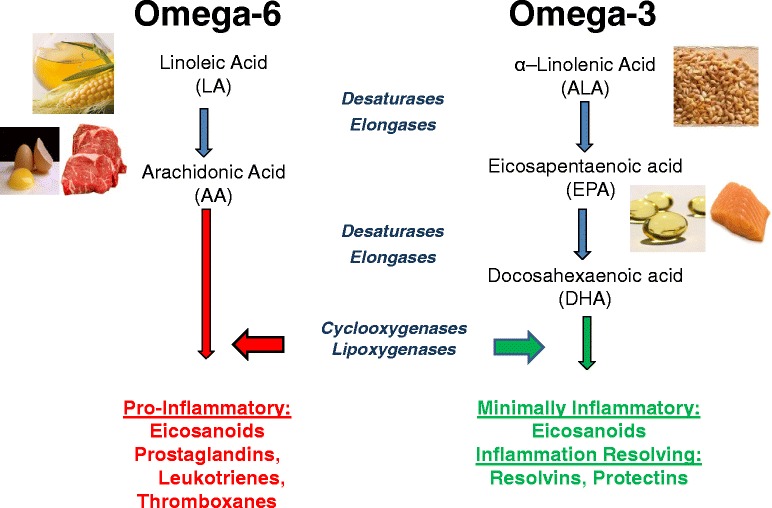
Dietary sources and general metabolic pathway for omega-6 and omega-3 polyunsaturated fatty acids, leading to proinflammatory and anti-inflammatory products respectively.

Omega-3 and omega-6 fatty acids are a group of essential polyunsaturated fatty acids (PUFAs) that play important roles in cell membrane structure, fluidity, and cell signaling [13]. The designation 3 or 6 is structural, referring to the double bond on the third or sixth carbon respectively from the methyl group [13]. The most abundant dietary PUFAs are the short-chain omega-3 alpha linolenic acid (ALA) and the omega-6 linoleic acid (LA), most often ingested as plant oils. The longer chain omega-3 PUFAs EPA and DHA, commonly referred to as marine fatty acids, are most efficiently obtained from fatty cold water fish such as salmon, whereas the long-chain omega-6 fatty acid AA is obtained most efficiently from eggs, poultry, and meat [24-26] (see Figure 1). Unless EPA, DHA, and AA are directly ingested, they must be derived from ALA and LA, respectively. In general, the desaturases and elongases have a greater affinity for ALA than LA but, due to the general 10-fold higher intake of LA, generally more AA than EPA and DHA is formed [24].

**Figure 2:**
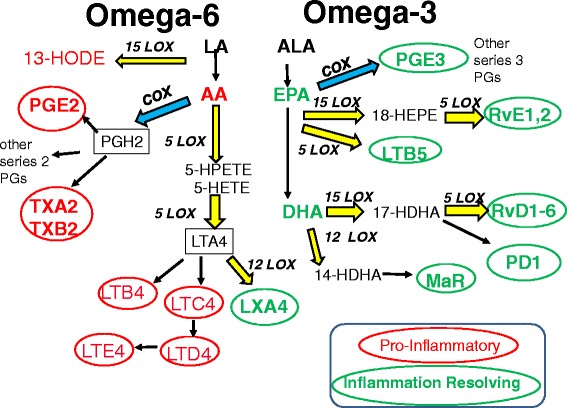
Metabolic pathways for omega-6 and omega-3 fatty acids that result in a variety of inflammation mediators and cell function effectors. Proinflammatory (red) and anti-inflammatory or less inflammatory (green) molecules are denoted within ellipses. Other molecules are indicated that are likely to promote (red) or repress (green) neoplastic processes. Cyclooxygenase (blue) and lipoxygenase (yellow) enzymatic processes are indicated. AA, arachidonic acid; ALA, alpha linolenic acid; COX, cyclooxygenase; DHA, docosahexaenoic acid; EPA, eicosapentaenoic acid; HDHA, hydroxydocosahexaenoic acid; HEPE, hydroxyeicosapentaenoic acid; HETE, hydroxyeicosatetraenoic acid; HODE, hydroxyoctadecadienoic acid; HPETE, hydroperoxyeicosatetraenoic acid; LA, linoleic acid; LOX, lipoxygenase; LT, leukotriene; LX, lipoxin; HODE, hydroxyoctadecadienoic acid; HX, hepoxilin; MaR, maresin; PD1, protectin D1; PG, prostaglandin; Rv, resolvin; TX, thromboxane.

Whether ingested or synthesized, PUFAs are either oxidized for fuel, stored in triacylglycerol, taken up in phospholipid membranes for eventual use as substrates by cyclooxygenase (COX) and lipoxygenase (LOX) enzymes, or used as ligands for G receptors [26]. Neither LA nor ALA is readily converted to bioactive lipid products due to low uptake into phospholipid membranes. However, 5 to 10% of both LA and ALA can be converted to the longer chain PUFAs that are readily taken up in phospholipid membranes and form the substrates for conversion to bioactive lipid products by COX and LOX enzymes [26] (see Figure 2).

The omega-6 PUFA AA and its derivatives are important in a diverse set of physiologic functions including initiation and sustainment of inflammation (for example, T-cell and monocyte activation, chemotaxis), platelet aggregation, endothelial adhesion molecules, ovulation, parturition, and muscle strength. The omega-3 fatty acids EPA and DHA and their derivatives are important for retina and brain development, cognitive function, and in the production of minimally inflammatory eicosanoids as well inflammation resolving mediators termed resolvins and various tissue protectins [20,22]. Although most of the bioactive lipid mediators of interest are a result of COX and LOX enzyme activity on the long-chain PUFAs EPA, DHA, and AA, 15-LOX acts on the short chain LA to form 13(S)-hydroxyoctadecadienoic acid, which is probably carcinogenic and is known to increase mammary tumor proliferation [22] (see Figure 2). EPA and DHA compete with AA as substrates for COX and LOX enzymes although EPA is a poorer substrate than AA, at least for COX [24].

Upon inflammatory stimulus, the enzyme phospholipase A2 releases AA from phospholipid membranes of monocytes and predominantly proinflammatory derivatives are produced (Figure 2). COX-1 and COX-2 enzymes are responsible for AA-derived prostaglandin E_2_ and other series-two prostaglandins and thromboxanes [15,24]. 5-LOX, 12-LOX, and 15-LOX are responsible for generation of the series-four leukotrienes and lipoxins. Leukotrienes have chemotactic and other effects on inflammatory cells.

In general, the action of COX and LOX enzymes on the omega-3 fatty acids EPA and DHA is to produce eicosanoids with less affinity for the corresponding receptors as well as resolvins that block inflammatory cell recruitment and promote phagocytosis. The net effect if EPA and DHA are present in sufficient amounts relative to AA is anti-inflammatory or inflammation resolving. The action of COX on EPA gives rise to the series-three prostaglandins and thromboxanes, whereas actions of 5-LOX and 15-LOX ultimately produce the series-five leukotrienes and resolvins. LOX enzymes are also responsible for the DHA-derived resolvins and eventual production of neuroprotectins [15,26] (see Figure 2).

## Recommended and average intakes and sources of omega-3 and omega-6 fatty acids

No dietary reference intake has been established for EPA and DHA. Although the dietary reference intake for omega-3 ALA of 1.1 g/day for women [27] is achieved by the average intake in the United States of 1.3 g/day, this is only about 1/10 of the 13 to 15 g daily intake of omega-6 LA [28].

Given the general health benefits increasingly recognized for EPA and DHA, many organizations have made recommendations for direct intake of 200 to 500 mg/day EPA + DHA for adult general health in the form of fish or fish oil, krill oil, or algae oil supplements (see Table 1). Average intakes of EPA and DHA combined, usually from fish or supplements, is ~100 mg/day or 0.1 to 0.2% of calories. Intake of AA is ~250 mg/day, generally from eggs, meat, and poultry [21,25,28].

**Table 1:** **Recommended intakes of EPA + DHA by cohort and organization**

**Cohort**	**Source**	**Daily recommendation**
General health		
Adults	US Department of Agriculture	≥250 mg
Adults	European Food Safety Agency	≥250 mg
Adults	World Health Organization:	≥250 mg
Adults	Academy of Nutrition and Dietetics	≥500 mg
Adults without CHD	American Heart Association	∼500 mg (fatty fish ≥ 2 times/week)
Adults	International Society for the Study of Fatty Acids and Lipids	≥500 mg
Pregnancy		
Pregnant/lactating	International Society for the Study of Fatty Acids and Lipids	≥500 mg (≥300 mg DHA)
Pregnant/lactating	European Food Safety Agency	≥250 mg (100 to 200 mg DHA)
Heart disease and inflammatory disorders		
CHD	American Heart Association	∼1 g
Patients with high TG	American Heart Association	2 to 4 g
Generally viewed as safe upper limit		
Population	US Food and Drug Administration	≤3 g EPA + DHA
Population	European Safety Authority	5 g EPA + DHA

CHD, coronary heart disease; DHA, docosahexaenoic acid; EPA, eicosapentaenoic acid; TG, triglyceride.

The omega-3 fatty acid intakes recommended for healthy individuals are not likely to be effective in chronic inflammatory conditions, given the level of omega-6 fatty acids in our diets [15,23,25,28]. If the ratio of EPA + DHA to AA in blood or tissue is the key factor [25,28], an intake of ~2 to 3 g/day combined EPA and DHA, or at least 2% of calories, is likely to be needed to result in a tissue level ratio of EPA + DHA to AA that approaches or exceeds unity. Doses generally exceeding 2 g/day combined EPA + DHA are needed to reduce prostaglandin E_2_ levels [26] and doses of 3 to 3.5 g/day combined EPA + DHA are most often used in the treatment of hypertriglyceridemia or inflammatory disorders such as rheumatoid arthritis [15,29]. No tolerable upper limit has been set for EPA and DHA, although the US Food and Drug Administration recognizes doses of up to 3 g/day as safe and the European Safety Union up to 5 g/day as safe [30]. Side effects of fish oil supplements or EPA + DHA ethyl esters include fishy burps, dyspepsia, gas, and diarrhea [15,29].

Primary sources of EPA and DHA are fish and supplements, which vary dramatically in their content. DHA is generally present in equal or higher amounts than EPA in seafood but the total amount of EPA and DHA as well as the ratios of EPA to DHA vary by supplement, and in many over-the-counter supplements EPA may be almost twice as high as DHA (see Tables 2 and 3). Fatty cold water fish such as salmon, herring, and mackerel have the highest levels of DHA and EPA, with lower levels in shellfish and many popular freshwater fish. A total of 2.4 g EPA + DHA can be obtained from a 4 oz (114 g) serving of wild Atlantic salmon but one would have to eat 8 oz (227 g) canned pink salmon, 1 lb (0.45 kg) halibut, or 5 lb (2.27 kg) shrimp to obtain the same amount (see Table 2) [31].

**Table 2:** **Dietary sources of EPA + DHA**

**EPA + DHA (mg)**	**Source (4 oz or 114 g serving)**
2,400	Salmon (Atlantic), herring (Pacific)
2,290	Herring (Atlantic)
2,060	Mackerel (Pacific and jack)
1,940	Salmon (chinook)
1,830	Whitefish
1,710	Tuna (bluefin)
1,560	Oysters (cooked)
1,490	Salmon (coho, farmed)
1,370	Mackerel (Atlantic), trout (farmed, rainbow)
1,190	Pink salmon canned
1,110	Bass (striped)
1,030	Trout (mixed species)
980	Tuna albacore canned
870	Bass (sea bass, freshwater)
540	Halibut (Atlantic and Pacific)
470	King crab
340	Flounder/sole
320	Tuna (yellowfin), Cod (Pacific)
270	Catfish wild
220	Lobster
180	Cod (Atlantic)
150	Tilapia
120	Shrimp
100	Catfish (farm)

DHA, docosahexaenoic acid; EPA, eicosapentaenoic acid.

**Table 3:** **Amounts of EPA and DHA in commonly available supplements**

**Brand/product**	**EPA (mg)**	**DHA (mg)**	**Comment**
Cardiotabs omega-3 enteric-coated	270	705	
Cardiotabs omega-3 extra strength + vitamin D3	400	800	
Cardiotabs ultra strength liquid omega-3 + vitamin D3 (per 5 ml)	610	1570	
DSM MEG-3® ethyl ester 1,000 mg capsules	420	210	As ethyl esters
GlaxoSmithKline Lovaza™	445	385	As ethyl esters
GNC ultra triple strength omega 1560 EPA & DHA	719	281	
GNC Now® ultra omega-3	500	250	
GNC Natrol® omega-3 fish oil 1,200 mg	216	144	
GNC Triple Strength Omega Complex	647	253	
GNC Triple Strength Fish Oil 1500	540	360	
Kirkland Signature™ omega-3 fish oil	410	274	As ethyl esters
NatureMade Fish Oil 1,200 mg	180	120	
NatureMade Ultra Omega-3 Fish Oil 1,400 mg	683	252	
Nordic Naturals Ultimate Omega D3, 1,000 mg, soft gels, lemon	650	450	
Puritan’s Pride Triple Omega 3-6-9 fish and flax oils	398 to 450	255 to 300	

DHA, docosahexaenoic acid; EPA, eicosapentaenoic acid.

## How might EPA and DHA act to prevent breast cancer? Preclinical mechanistic studies

Most of the work assessing how EPA and DHA might work to reduce breast cancer risk has been performed in *in vitro* or in transgenic mouse models and is far from conclusive. However, the predominant mechanisms are thought to be: a reduction in proinflammatory eicosanoids and an increase in inflammation-resolving derivatives as detailed previously (Figure 2); a reduction in oncogenic protein signaling through disruption of plasma membrane lipid rafts; a reduction in cytokine production; and an increase in apoptosis following activation of plasma membrane GRP120 protein receptor, which along with activation of peroxisome proliferator-activated receptor gamma blocks nuclear factor-κB translocation to the nucleus [8,9].

EPA and DHA disrupt lipid rafts, sphingolipid/cholesterol-enriched microdomains of plasma membranes that optimize signaling by concentrating proteins. Lipid rafts are particularly important for several tyrosine kinase receptors, and reduction in epidermal growth factor receptor and human epidermal growth factor-2 receptor level and activation has been demonstrated in transformed and malignant cells [10-12]. A decrease in epidermal growth factor receptor and human epidermal growth factor-2 signaling would be expected to reduce proliferation, and a decrease in Ki-67 has indeed been observed in benign and malignant mammary tissue after EPA and DHA supplementation in most preclinical models [32-35].

Nuclear factor-κB nuclear translocation and signaling is reduced via the agonist effects of EPA and DHA on peroxisome proliferator-activated receptor gamma as well as interaction with the G protein receptor GPR120, with expected reduction in inhibitors of apoptosis as well as cytokines, adhesion molecules, and metalloproteases [9]. Additional preclinical studies suggest that EPA and DHA increase expression of BRCA1/2, phosphatase and tensin homolog (PTEN), and other proteins associated with cell cycle control and DNA repair [32,36,37].

## Preclinical models of mammary cancer prevention

Studies in rodent models find that increasing the ratio of total omega-3:omega-6 in feed to >1 (usually with EPA + DHA between 8 and 25% of calories) reduces mammary cancer incidence and multiplicity by 20 to 35% [22,32,37-41]*.* Reductions in tumor incidence have been observed in the estrogen receptor-negative MMTV-HER-2/neu transgenic mice [39,40], the estrogen receptor-positive NMU rat model [32,37], and the estrogen receptor-positive DMBA rat model [41-43]. The minimum dose of marine omega-3 fatty acids for effect is not clear and may vary by animal model, source of EPA and DHA (fish oil versus ethyl esters), and total amount and type of fat in the diet. Other important experimental conditions include when in an animal’s lifespan supplementation is started (younger may be more protective than older), and whether agent is added to feed or administered by gavage as omega-3 fatty acids are readily oxidized once exposed to light [38]. Several preclinical studies suggest that EPA/DHA supplementation may be most optimal for prevention of estrogen receptor-positive breast cancer when used with another chemoprevention agent such as vitamin D [41], a selective estrogen receptor modulator [43], or celecoxib [42].

## Human studies

Results of case–control and cohort studies have to date been variable, probably reflecting the heterogeneity of cohorts, methods used to assess omega-3 and omega-6 exposure, time from exposure when measures were taken, dose, and response endpoint.

### Pharmacodynamics

Omega-3 and omega 6 fatty acids are incorporated at different rates in different tissues and tissue components. Levels as a percentage of total fatty acids vary tremendously between tissues/organs although, with supplementation, levels of EPA and DHA rise in a fairly proportional manner [44]. Substantial increases in monocyte membrane DHA and EPA and decreases in monocyte AA may be seen as early as 1 week after beginning supplementation and do not change dramatically over the ensuing several weeks [9,26]. The time to EPA maximum uptake is ~2 weeks in plasma triglycerides, 3 weeks in serum cholesterol esters, ~2 months in red blood cells (RBCs), and >12 months for most types of adipose tissue. The highest levels of EPA and DHA in the blood are generally in the RBC membranes (RBC phospholipids), plasma phospholipids, and cholesterol esters and platelets, although mononuclear cells also contain appreciable amounts [45]. The concentration of EPA and DHA in subcutaneous or breast adipose is 1/10 or less of that in the blood compartments [44]. DHA is generally much higher than EPA in most body organs, including the brain and retina, but its incorporation into RBCs lags behind EPA [26,28,29,46]. Women generally have higher levels of EPA and DHA than men following equivalent dosing, and older women have higher levels than younger women [45]. Individuals who take fish oil supplements tend to take them daily whereas consumption of fish may be more intermittent. Browning and colleagues determined in a 12-month study of adults taking identical weekly doses of EPA + DHA that those taking continuous daily doses had higher EPA and DHA levels in monocytes and platelets than those taking intermittent doses [47].

For clinical trials, chronic exposure is generally assessed by measuring EPA, DHA, and AA RBC phospholipids, although some investigators feel that monocyte or platelet phospholipid measures are superior to those in RBCs [29].

### Case–control studies

Results of case–control studies, particularly when questionnaires are used as the primary measure of exposure, are mixed, probably reflective of the accuracy of recall and food frequency questionnaires in estimating dietary intake. There is no significant association between total fish intake and breast cancer particularly in populations where total fish and fatty fish consumption tends to be low [48,49]. EPA and DHA content varies tremendously by type of fish, which may not be well specified in the questionnaires. However, two case–control studies (one from Mexico and another from the United States) using dietary recall instruments suggest breast cancer risk reduction in premenopausal women with higher intakes of omega-3 fatty acids from diet and supplements [50,51].

Measurement of the fatty acid composition in blood cell membranes (phospholipids) and adipose is thought to be a good indicator of chronic exposure to omega-3 and omega-6 fatty acids and thus avoids some of the problems with dietary recall. A nested case–control study within a prospective cohort of women in Shanghai China, a population with relatively high fish intake, found that total omega-3 fatty acids and EPA in red cells were associated with significantly lower risk of proliferative breast disease and breast cancer [52,53]. Similar findings were reported in a Japanese cohort where total omega-3, EPA, and DHA in red cells was inversely associated with breast cancer risk [54]. Another case–control study suggested reduced risk of breast cancer with higher ratios of omega-3 to omega-6 in breast adipose [55]. No association was reported between the risk biomarker mammographic breast density and omega-3 fatty acids [56].

## Prospective cohort studies of omega-3 fatty acids and breast cancer risk

A meta-analysis of 16 prospective cohort studies examining marine omega-3 intake suggests a reduction in breast cancer risk when individuals with highest intakes are compared with those with lowest intakes of marine PUFA (EPA, docosapentaenoic acid, and DHA) in the diet or the diet plus supplements [57]. The method for assessment of marine PUFA exposure varied from dietary questionnaire to blood or tissue n-3 PUFA assessment. Overall the relative risk for highest exposure was 0.86 (95% confidence interval, 0.97 to 1.03). The affect appeared strongest for marine PUFA in postmenopausal women but there were fewer premenopausal women studied [57]. In three of the largest studies – the Singapore Chinese Health Study [58], the Japanese Collaborative Cohort Study [59], and the Vitamins and Lifestyle (VITAL) study from western Washington state [60] – there was a significant reduction in relative risk in the individual trials ranging from 31 to 50%. Current use of fish oil supplements (generally 300 mg EPA + DHA or more per capsule) in the VITAL trial in women aged >50 years was associated with a 32% reduction in risk of breast cancer (hazard ratio, 0.68; 95% confidence interval, 0.50 to 0.92) [60,61].

Eight studies were available for dose–response analysis, which showed that a 0.1 g/day increment and/or 0.1% of energy intake increments were associated with a 5% reduction in breast cancer risk [57]. In this same meta-analysis no association was observed between total fish intake, total PUFA or ALA (the shorter chain omega-3 fatty acid) intake and breast cancer risk [57].

A recent meta-analysis combined six prospective nested case–control studies and five cohort studies in which the omega-3:omega-6 intake ratio and/or omega-3:omega-6 ratio in serum phospholipids was known. There were over 274,000 women, and more than 8,300 breast cancer events. Their conclusions were that each 1/10 increment in the dietary n-3:n-6 ratio was associated with a 6% reduction in breast cancer risk, and amongst US subjects each 1/10 increment in the serum n-3:n-6 phospholipid ratio was associated with a 27% reduction in breast cancer risk [49].

### Interventional studies for primary prevention of breast cancer

Although one is not likely to achieve EPA + DHA intake in human trials at the same percentage of calories as in animal prevention trials, doses of EPA and DHA ethyl esters up to ~7 g/day given to healthy women are well tolerated [62]. A dose of 3.4 g/day DHA + EPA ethyl esters, providing ~ 2% of calories, is US Food and Drug Administration approved for treatment of hypertriglyceridemia. Importantly, this dose should produce an EPA + DHA:AA ratio approaching equivalence and thus provide an anti-inflammatory effect.

Human studies in healthy individuals show little effect of marine PUFA on blood inflammatory biomarkers, although a recent randomized trial in healthy young adults given 0, 300, 600, 900, or 1,800 mg/day EPA + DHA for 5 months showed a marginal decrease in serum tumor necrosis factor alpha (*P* = 0.08) but no change in interleukin-6 [63].

Human studies in inflammatory disorders show little evidence of a systemic anti-inflammatory effect such as reduction of cytokines or prostaglandin E_2_ levels with doses of combined EPA + DHA less than ~3.5 g/day and/or EPA-alone doses <2.7 g/day [26,64]. However, experts in this area suggest that systemic measures of cytokines in inflammatory conditions are likely to be insensitive compared with measuring conditions in the tissue of interest [65].

Signori and colleagues are conducting a trial of raloxifene 30 mg, raloxifene 60 mg, Lovaza™ (GlaxoSmithKline) 4 g, Lovaza™ 4 g + raloxifene 30 mg, or no intervention in postmenopausal women with >25% breast density. No change with Lovaza™ has been found in the first 46 women in secondary endpoint blood risk biomarkers such as insulin-like growth factor I and insulin-like growth factor-binding protein 3 or the inflammatory marker high-sensitivity C-reactive protein [66].

We have completed separate pilot studies of 3.4 g/day EPA + DHA ethyl esters (4 g Lovaza™) administered for 6 months to explore effects on benign breast tissue risk biomarkers for breast cancer in premenopausal and postmenopausal women at increased risk for breast cancer. Favorable modulation of several tissue risk biomarkers for breast cancer was observed [67,68].

A study of particular interest is the ongoing VITAL trial (NCT01169259) which aims to randomize over 28,000 men and women to vitamin D3 (2,000 IU/day), omega-3 fatty acids (840 mg EPA + DHA), both, or none, with a primary outcome of reduction in risk for cancer, stroke, and other diseases. Eligible women must be age 55 years and over.

## Omega-3 fatty acids and breast cancer survivorship

There is also interest in EPA and DHA for improvement of outcomes after a diagnosis of breast cancer. Breast cancer recurrence, cardiovascular events, weight gain and obesity, bone density loss, and chemotherapy-associated cognitive impairment and peripheral neuropathy are common concerns during the survivorship period. Although there is little in the way of definitive interventional trials, we will review here some of the more interesting preliminary results.

### EPA and DHA and reduction of breast cancer recurrence

Higher intakes of EPA and DHA from dietary sources were reported to be associated with a 25% reduction in breast cancer recurrence and improved overall mortality in a large cohort of over 3,000 women with early stage breast cancer followed for a median of 7 years [69]. One reason for this observation may be enhancement of at least some types of chemotherapeutic cytotoxicity, which has been reported for concomitant administration of DHA with anthracyclines [70,71]. This enhanced cytotoxicity probably results from alteration in membrane lipid rafts, which increases surface expression and clustering of the death receptor CD95 in mammary cancer cell lines treated with EPA and DHA and doxorubicin [72]. Improved outcome with DHA added to chemotherapy in a small phase II trial has been reported in metastatic breast cancer patients [73]. This observation raises the question of whether cardiac toxicity might also be increased by adding EPA or DHA to anthracyclines, but this does not appear to be the case at least in rats [74].

### EPA and DHA to reduce cardiac events

Cardiac events are the second most common cause of mortality in women with breast cancer, and the most common cause of death for women with stage I breast cancer over the age of 65. EPA and DHA reduce triglycerides and platelet aggregation and are thought to have an anti-arrhythmic effect. EPA and DHA supplementation have been noted to be associated with reduced cardiac deaths in the general population [75,76]. A highly purified prescription strength form of ~3.4 g/day EPA and DHA (Lovaza™, formerly omacor, 4 g/day) is US Food and Drug Administration approved for treatment of hypertriglyceridemia and has been shown to reduce triglycerides and nonhigh-density lipoproteins to a greater extent than a statin alone in individuals with mixed dyslipidemia and triglycerides >200 mg/dl [77]. This highly purified prescription formulation has also been shown to reduce cardiac events and mortality in individuals with a prior myocardial infarction at lower doses of 1 g/day [77]. However, a recent secondary prevention trial with 1 g/day EPA and DHA compared with 1 g/day olive oil did not show any cardioprotective effect [78]. A recent meta-analysis of EPA and DHA in moderate doses also showed no benefit [79]. The cause of these discrepancies is open to speculation. Possibilities include the following: 1) a lack of additional benefit for EPA + DHA in women with cardiac disease already on optimal medical management; 2) the placebo, often olive oil, may also have cardiovascular benefit; 3) or the highly purified forms of EPA + DHA may have special properties such as lower reactive oxygen species than less purified forms of fish oil [80]. Trials such as the VITAL trial in women without a prior history of heart disease will be of great interest.

### EPA and DHA to reduce bone density loss and arthralgias

Loss of bone density and increased fracture rate are a side effect of premature menopause caused by cytotoxic chemotherapy or surgical ovarian ablation in premenopausal women or use of aromatase inhibitors in postmenopausal women. EPA and DHA probably inhibit RANK ligand and osteoclast formation [81]. A small randomized pilot trial suggests that 3 g/day EPA and DHA inhibits bone reabsorption in individuals taking aromatase inhibitors [82]. The anti-inflammatory activity and beneficial effects of EPA and DHA on rheumatoid arthritis have led to a clinical trial of high-dose EPA and DHA versus placebo in women who have aromatase inhibitor-induced arthralgias. This cooperative group study of 262 women has been reported in abstract form and no benefit was observed [83]. A small randomized trial of omega-3 fatty acids to protect against taxane-induced neuropathy suggests benefit [84] and further studies are needed.

### EPA and DHA to prevent insulin resistance and sarcopenic weight gain

EPA and DHA help prevent obesity and insulin resistance particularly in animal models fed a high-fat diet [85,86], but effects in humans have yet to be proven. Sarcopenic weight gain is common during adjuvant chemotherapy for breast cancer. The anabolic effects of EPA and DHA might help reduce muscle mass loss and weight gain during treatment and weight gain following diagnosis, but studies in this area have yet to be conducted [87,88]. Results of the Muscle Mass, Omega-3, Diet, Exercise and Lifestyle (MODEL) trial in healthy individuals aged >70 years examining the effects of 90 minutes of exercise weekly, vitamin D3 (2,000 IU/day) or 1 g EPA and DHA daily are awaited with interest [89].

### EPA and DHA and cognition

Cognitive abnormalities are observed in 20 to 70% of women after chemotherapy depending on the agents used, intensity and duration of treatment, predisposing factors, and type and scoring of cognitive tests [90,91]. DHA is the most abundant PUFA in the brain and is involved in multiple functions including cell signaling, neurogenesis, neuroprotection, and learning and memory [18]. A number of epidemiologic studies show a 40 to 50% reduction in risk of multicause dementia with increased dietary intake of DHA or increased blood levels of DHA [19]. In meta-analyses, DHA supplementation improves attention, processing speed and immediate recall, learning, and memory in individuals with cognitive impairment without dementia but not in those with dementia [92,93]. Probable mechanisms include suppression of oxidative stress [94], decreases in proinflammatory lipid derivatives from AA, an increase in inflammation resolving and protective lipid derivatives, enhanced production of neurotransmitters [95], and reduced production and accumulation of amyloid B peptide toxin [19]. Doses of DHA administered as supplements for cognitive improvement are generally in the range of 1,800 mg/day. Studies utilizing DHA or DHA + EPA as a neuroprotectant during chemotherapy are needed.

## Conclusion

The inflammation-resolving properties and favorable effects of EPA and DHA on oncogenic proteins, as well as on the cardiovascular, bone, and central nervous system, make them excellent candidates for primary and secondary breast cancer prevention trials for individuals at increased risk as well as breast cancer survivors. Interventional trials in these cohorts are ongoing.

## Abbreviations

AA: Arachidonic acid
ALA: Alpha linolenic acid
COX: Cyclooxygenase
DHA: Docosahexaenoic acid
EPA: Eicosapentaenoic acid
LA: Linoleic acid
LOX: Lipoxygenase
PUFA: Polyunsaturated fatty acid
RBC: Red blood cell
VITAL: Vitamins and lifestyle

## References

[1] Howe LR, Subbaramaiah K, Hudis CA, Dannenberg AJ (2013). Molecular pathways: adipose inflammation as a mediator of obesity-associated cancer. Clin Cancer Res.

[2] Baumgarten SC, Minireview FJ (2012). Inflammation: an instigator of more aggressive estrogen receptor (ER) positive breast cancers. Mol Endocrinol.

[3] Hussein MR, Hassan HI (2006). Analysis of the mononuclear inflammatory cell infiltrate in the normal breast, benign proliferative breast disease, in situ and infiltrating ductal breast carcinomas: preliminary observations. J Clin Pathol.

[4] Pollard J (2008). Macrophages define the invasive microenvironment in breast cancer. J Leukoc Biol.

[5] McDermott RS, Beuvon F, Pauly M, Pallud C, Vincent-Salomon A, Mosseri V (2002). Tumor antigens and antigen-presenting capacity in breast cancer. Pathobiology.

[6] Greenberg AS, Obin MS (2006). Obesity and the role of adipose tissue in inflammation and metabolism. Am J Clin Nutr.

[7] Weylandt KH, Chiu CY, Gomolka B, Waechter SF, Wiedenmann B (2012). Omega-3 fatty acids and their lipid mediators: towards an understanding of resolvin and protectin formation. Prostaglandins Other Lipid Mediat.

[8] Turk HF, Chapkin RS (2013). Membrane lipid raft organization is uniquely modified by n-3 polyunsaturated fatty acids. Prostaglandins Leukot Essent Fatty Acids.

[9] Calder PC (2013). n-3 fatty acids, inflammation and immunity: new mechanisms to explain old actions. Proc Nutr Soc.

[10] Ravacci GR, Brentani MM, Tortelli T, Torrinhas RS, Saldanha T, Torres EA (2013). Lipid raft disruption by docosahexaenoic acid induces apoptosis in transformed human mammary luminal epithelial cells harboring HER-2 overexpression. J Nutr Biochem.

[11] Lee EJ, Yun UJ, Koo KH, Sung JY, Shim J, Ye SK (1841). Down-regulation of lipid raft-associated onco-proteins via cholesterol-dependent lipid raft internalization in docosahexaenoic acid-induced apoptosis. Biochim Biophys Acta.

[12] Rogers KR, Kikawa KD, Mouradian M, Hernandez K, McKinnon KM, Ahwah SM (2010). Docosahexaenoic acid alters epidermal growth factor receptor-related signaling by disrupting its lipid raft association. Carcinogenesis.

[13] Calder PC (2011). Fatty acids and inflammation: the cutting edge between food and pharma. Eur J Pharmacol.

[14] Wen ZH, Su YC, Lai PL, Zhang Y, Xu YF, Zhao A (2013). Critical role of arachidonic acid-activated mTOR signaling in breast carcinogenesis and angiogenesis. Oncogene.

[15] Yates CM, Calder PC, Ed RG (2014). Pharmacology and therapeutics of omega-3 polyunsaturated fatty acids in chronic inflammatory disease. Pharmacol Ther.

[16] Simopoulos AP (2006). Evolutionary aspects of diet, the omega-6/omega-3 ratio and genetic variation: nutritional implications for chronic diseases. Biomed Pharmacother.

[17] Friborg JT, Melbye M (2008). Cancer patterns in Inuit populations. Lancet Oncol.

[18] Su H-M (2010). Mechanisms of n-3 fatty acid-mediated development and maintenance of learning memory performance neuroprotection. J Nutr Biochem.

[19] Cole GM, Ma QL, Frautschy SA (2009). Omega-3 fatty acids and dementia. Prostaglandins Leukot Essent Fatty Acids.

[20] Simopoulos AP (1999). Evolutionary aspects of omega-3 fatty acids in the food supply. Prostaglandins Leukot Essent Fatty Acids.

[21] Blasbalg TL, Hibbeln JR, Ramsden CE, Majchrzak SF, Rawlings RR (2011). Changes in consumption of omega-3 and omega-6 fatty acids in the United States during the 20th century. Am J Clin Nutr.

[22] Rose D, Connolly J (1999). Omega-3 fatty acids as cancer chemopreventive agents. Pharmacol Ther.

[23] Simonsen N, van't Veer P, Strain JJ, Martin-Moreno JM, Huttunen JK, Navajas JF (1998). EURAMIC study. European Community Multicenter Study on Antioxidants, Myocardial Infarction, and Breast Cancer. Am J Epidemiol.

[24] Russo GL (2009). Dietary n-6 and n-3 polyunsaturated fatty acids: from biochemistry to clinical implications in cardiovascular prevention. Biochem Pharmacol.

[25] Brenna JT, Salem N, Sinclair AJ, Cunnane SC (2009). International Society for the Study of Fatty Acids and Lipids, ISSFAL, alpha-linolenic acid supplementation and conversion to n-3 long-chain polyunsaturated fatty acids in humans. Prostaglandins Leukot Essent Fatty Acids.

[26] Calder PC (2013). Omega-3 polyunsaturated fatty acids and inflammatory processes: nutrition or pharmacology?. Br J Clin Pharmacol.

[27] Flock MR, Harris WS, Kris-Etherton PM (2013). Long-chain omega-3 fatty acids: time to establish a dietary reference intake. Nutr Rev.

[28] Arterburn LM, Hall EB, Oken H (2006). Distribution, interconversion, and dose response of n-3 fatty acids in humans. Am J Clin Nutr.

[29] Browning LM, Walker CG, Mander AP, West AL, Madden J, Gambell JM (2012). Incorporation of eicosapentaenoic and docosahexaenoic acids into lipid pools when given as supplements providing doses equivalent to typical intakes of oily fish. Am J Clin Nutr.

[30] European Food Safety Authority (2012). Scientific Opinion on the Tolerable Upper Intake Level of eicosapentaenoic acid (EPA), docosahexaenoic acid (DHA) and docosapentaenoic acid (DPA). EFSA J.

[31] Addendum A. EPA and DHA Content of Fish Species. United States Department of Agriculture. 2004. www.health.gov/dietaryguidelines/dga2005/report/HTML/G2_Analyses.htm# omegafish. Accessed 10 Mar 2015.

[32] Jiang W, Zhu Z, McGinley JN, El Bayoumy K, Manni A, Thompson HJ (2012). Identification of a molecular signature underlying inhibition of mammary carcinoma growth by dietary N-3 fatty acids. Cancer Res.

[33] Manni A, Richie JP, Xu H, Washington S, Aliaga C, Cooper TK (2011). Effects of fish oil and tamoxifen on preneoplastic lesion development and biomarkers of oxidative stress in the early stages of N-methyl-N-nitrosourea-induced rat mammary carcinogenesis. Int J Oncol.

[34] Manna S, Janarthan M, Ghosh B, Rana B, Rana A, Chatterjee M (2010). Fish oil regulates cell proliferation, protect DNA damages and decrease HER-2/neu and c-Myc protein expression in rat mammary carcinogenesis. Clin Nutr.

[35] Yee LD, Agarwal D, Rosol TJ, Lehman A, Tian M, Hatton J (2013). The inhibition of early stages of HER-2/neu-mediated mammary carcinogenesis by dietary n-3 PUFAs. Mol Nutr Food Res.

[36] Bernard-Gallon D, Vissac-Sabatier C, Antoine-Vincent D, Rio PG, Maurizis JC, Fustier P (2002). Differential effects of n-3 and n-6 polyunsaturated fatty acids on BRCA1 and BRCA2 gene expression in breast cell lines. Br J Nutr.

[37] Jourdan M-L, Mahéo K, Barascu A, Goupille C, De Latour MP, Bougnoux P (2007). Increased BRCA1 protein in mammary tumours of rats fed marine ω-3 fatty acids. Oncol Rep.

[38] Signori C, El-Bayoumy K, Russo J, Thompson HJ, Richie JP, Hartman TJ (2011). Chemoprevention of breast cancer by fish oil in preclinical models: trials and tribulations. Cancer Res.

[39] MacLennan MB (2013). Mammary tumor development is directly inhibited by lifelong n-3 polyunsaturated fatty acids. J Nutr Biochem.

[40] Yee LD, Young DC, Rosol TJ, VanBuskirk AM, Clinton SK (2005). Dietary (n-3) polyunsaturated fatty acids inhibit HER-2/neu induced breast cancer in mice independently of the PPARγ ligand rosiglitazone. J Nutr.

[41] Chatterjee M, Janarthan M, Manivannan R, Rana A, Chatterjee M (2010). Combinatorial effect of fish oil (Maxepa) and 1alpha,25-dihydroxyvitamin D(3) in the chemoprevention of DMBA-induced mammary carcinogenesis in rats. Chem Biol Interact.

[42] Negi AK, Kansal S, Bhatnagar A, Agnihotri N (2013). Alteration in apoptosis and cell cycle by celecoxib and/or fish oil in 7,12-dimethyl benzene (α) anthracene-induced mammary carcinogenesis. Tumour Biol.

[43] Manni A, Richie JP, Xu H, Washington S, Aliaga C, Bruggeman R (2014). Influence of omega-3 fatty acids on tamoxifen-induced suppression of rat mammary carcinogenesis. Int J Cancer.

[44] Katan MB, Deslypere JP, van Birgelen AP, Penders M, Zegwaard M (1997). Kinetics of the incorporation of dietary fatty acids into serum cholesteryl esters, erythrocyte membranes, and adipose tissue: an 18-month controlled study. J Lipid Res.

[45] Walker CG, Browning LM, Mander AP, Madden J, West AL, Calder PC (2014). Age and sex differences in the incorporation of EPA and DHA into plasma fractions, cells and adipose tissue in humans. Br J Nutr.

[46] Kopecky J, Rossmeisl M, Flachs P, Kuda O, Brauner P, Jilkova Z (2009). n-3 PUFA: bioavailability and modulation of adipose tissue function. Proc Nutr Soc.

[47] Browning LM, Walker CG, Mander AP, West AL, Gambell J, Madden J (2014). Compared with daily, weekly n-3 PUFA intake affects the incorporation of eicosapentaenoic acid and docosahexaenoic acid into platelets and mononuclear cells in humans. J Nutr.

[48] Terry PD, Rohan TE, Wolk A (2003). Intakes of fish and marine fatty acids and the risks of cancers of the breast and prostate and of other hormone-related cancers: a review of the epidemiologic evidence. Am J Clin Nutr.

[49] Yang B, Ren XL, Fu YQ, Gao JL, Li D (2014). Ratio of n-3/n-6 PUFAs and risk of breast cancer: a meta-analysis of 274135 adult females from 11 independent prospective studies. BMC Cancer.

[50] Chajès V, Torres-Mejía G, Biessy C, Ortega-Olvera C, Angeles-Llerenas A, Ferrari P (2012). ω-3 and ω-6 polyunsaturated fatty acid intakes and the risk of breast cancer in Mexican women: impact of obesity status. Cancer Epidemiol Biomarkers Prev.

[51] Goodstine SL, Zheng T, Holford TR, Ward BA, Carter D, Owens PH (2003). Dietary (n-3)/(n-6) fatty acid ratio: possible relationship to premenopausal but not postmenopausal breast cancer risk in U.S. women. J Nutr.

[52] Shannon J, King IB, Moshofsky R, Lampe JW, Gao DL, Ray RM (2007). Erythrocyte fatty acids and breast cancer risk: a case–control study in Shanghai. China Am J Clin Nutr.

[53] Shannon J, King IB, Lampe JW, Gao DL, Ray RM, Lin MG (2009). Erythrocyte fatty acids and risk of proliferative and nonproliferative fibrocystic disease in women in Shanghai. China Am J Clin Nutr.

[54] Kuriki K, Hirose K, Wakai K, Matsuo K, Ito H, Suzuki T (2007). Breast cancer risk and erythrocyte compositions of n-3 highly unsaturated fatty acids in Japanese. Int J Cancer.

[55] Bagga D, Anders KH, Wang HJ, Glaspy JA (2002). Long-chain n-3-to-n-6 polyunsaturated fatty acid ratios in breast adipose tissue from women with and without breast cancer. Nutr Cancer.

[56] Hudson AG, Reeves KW, Modugno F, Wilson JW, Evans RW, Vogel VG (2013). Erythrocyte omega-6 and omega-3 fatty acids and mammographic breast density. Nutr Cancer.

[57] Zheng JS, Hu XJ, Zhao YM, Yang J, Li D (2013). Intake of fish and marine n-3 polyunsaturated fatty acids and risk of breast cancer: meta-analysis of data from 21 independent prospective cohort studies. BMJ.

[58] Gago-Dominguez M, Yuan JM, Sun CL, Lee HP, Yu MC (2003). Opposing effects of dietary n-3 and n-6 fatty acids on mammary carcinogenesis: the Singapore Chinese Health Study. Br J Cancer.

[59] Wakai K, Tamakoshi K, Date C, Fukui M, Suzuki S, Lin Y (2005). Dietary intakes of fat and fatty acids and risk of breast cancer: a prospective study in Japan. Cancer Sci.

[60] Brasky TM, Lampe JW, Potter JD, Patterson RE, White E (2010). Specialty supplements and breast cancer risk in the VITamins And Lifestyle (VITAL) Cohort. Cancer Epidemiol Biomarkers Prev.

[61] Kris-Etherton PM, Grieger JA, Etherton TD (2009). Dietary reference intakes for DHA and EPA. Prostaglandins Leukot Essent Fatty Acids.

[62] Yee LD, Lester JL, Cole RM, Richardson JR, Hsu JC, Li Y (2010). Omega-3 fatty acid supplements in women at high risk of breast cancer have dose-dependent effects on breast adipose tissue fatty acid composition. Am J Clin Nutr.

[63] Flock MR, Skulas-Ray AC, Harris WS, Gaugler TL, Fleming JA, Kris-Etherton PM (2014). Effects of supplemental long-chain omega-3 fatty acids and erythrocyte membrane fatty acid content on circulating inflammatory markers in a randomized controlled trial of healthy adults. Prostaglandins Leukot Essent Fatty Acids.

[64] Yusof HM, Cawood AL, Ding R, Williams JA, Napper FL, Shearman CP (2013). Limited impact of 2 g/day omega-3 fatty acid ethyl esters (Omacor®) on plasma lipids and inflammatory markers in patients awaiting carotid endarterectomy. Mar Drugs.

[65] Sijben JW, Calder PC (2007). Differential immunomodulation with long-chain n-3 PUFA in health and chronic disease. Proc Nutr Soc.

[66] Signori C, DuBrock C, Richie JP, Prokopczyk B, Demers LM, Hamilton C (2012). Administration of omega-3 fatty acids and raloxifene to women at high risk of breast cancer: interim feasibility and biomarkers analysis from a clinical trial. Eur J Clin Nutr.

[67] Fabian CJ, Kimler BF, Petroff BK, Zalles CM, Metheny T, Nydegger JL, et al. High dose omega-3 fatty acid supplementation modulates breast tissue biomarkers in post-menopausal women at high risk for development of breast cancer [abstract]. Cancer Res. 2013;73:P4-10-01. doi:10.1158/0008-5472.SABCS13-P4-10-01.

[68] Fabian CF, Kimler BF, Petroff BK, Zalles CM, Metheny T, Box JA, et al. High dose omega-3 fatty acid (FA) supplementation modulates breast tissue biomarkers in pre-menopausal women at high risk for development of breast cancer [abstract]. J Clin Oncol. 2013;31 Suppl:abstract 1515.

[69] Patterson RE (2011). Marine fatty acid intake is associated with breast cancer prognosis. J Nutr.

[70] Kang KS, Wang P, Yamabe N, Fukui M, Jay T, Zhu BT (2010). Docosahexaenoic acid induces apoptosis in MCF-7 cells in vitro and in vivo via reactive oxygen species formation and caspase 8 activation. PLoS One.

[71] Colas S, Mahéo K, Denis F, Goupille C, Hoinard C, Champeroux P (2006). Sensitization by dietary docosahexaenoic acid of rat mammary carcinoma to anthracycline: a role for tumor vascularization. Clin Cancer Res.

[72] Ewaschuk JB, Newell M, Field CJ (2012). Docosahexanoic acid improves chemotherapy efficacy by inducing CD95 translocation to lipid rafts in ER(−) breast cancer cells. Lipids.

[73] Bougnoux P, Hajjaji N, Ferrasson MN, Giraudeau B, Couet C, Le Floch O (2009). Improving outcome of chemotherapy of metastatic breast cancer by docosahexaenoic acid: a phase II trial. Br J Cancer.

[74] Germain E, Bonnet P, Aubourg L, Grangeponte MC, Chajès V, Bougnoux P (2003). Anthracycline-induced cardiac toxicity is not increased by dietary omega-3 fatty acids. Pharmacol Res.

[75] Calder PC, Yaqoob P (2012). Marine omega-3 fatty acids and coronary heart disease. Curr Opin Cardiol.

[76] Baum SJ, Kris-Etherton PM, Willett WC, Lichtenstein AH, Rudel LL, Maki KC (2012). Fatty acids in cardiovascular health and disease: a comprehensive update. J Clin Lipidol.

[77] Bays H (2006). Clinical overview of omacor: a concentrated formulation of omega-3 polyunsaturated fatty acids. Am J Cardiol.

[78] Bosch J, Gerstein HC, Dagenais GR, Díaz R, Dyal L, ORIGIN Trial Investigators (2012). n-3 fatty acids and cardiovascular outcomes in patients with dysglycemia. N Engl J Med.

[79] Rizos EC, Ntzani EE, Bika E, Kostapanos MS, Elisaf MS (2012). Association between omega-3 fatty acid supplementation and risk of major cardiovascular disease events: a systematic review and meta-analysis. JAMA.

[80] Harris WS, Shearer GC (2014). Omega-6 fatty acids and cardiovascular disease: friend or foe?. Circulation.

[81] Bonnet N, Somm E, Rosen CJ (2014). Diet and gene interactions influence the skeletal response to polyunsaturated fatty acids. Bone.

[82] Hutchins-Wiese HL, Picho K, Watkins BA, Li Y, Tannenbaum S, Claffey K (2014). High-dose eicosapentaenoic acid and docosahexaenoic acid supplementation reduces bone resorption in postmenopausal breast cancer survivors on aromatase inhibitors: a pilot study. Nutr Cancer.

[83] Hershman DL, Unger JM, Crew KD, Dakhil SR, Awad D, Greenlee H, et al. Omega-3 fatty acids for aromatase inhibitor-induced musculoskeletal symptoms in women with early-stage breast cancer (SWOG S0927) [abstract]. J Clin Oncol. 2014;32:5 s Suppl: abstract 9532.

[84] Ghoreishi Z, Esfahani A, Djazayeri A, Djalali M, Golestan B, Ayromlou H (2012). Omega-3 fatty acids are protective against paclitaxel-induced peripheral neuropathy: a randomized double-blind placebo controlled trial. BMC Cancer.

[85] Flock MR, Rogers CJ, Prabhu KS, Kris-Etherton PM (2013). Immunometabolic role of long-chain omega-3 fatty acids in obesity-induced inflammation. Diabetes Metab Res Rev.

[86] Fedor D, Kelley DS (2009). Prevention of insulin resistance by n-3 polyunsaturated fatty acids. Curr Opin Clin Nutr Metab Care.

[87] Buckley JD, Howe PRC (2010). Long-chain omega-3 polyunsaturated fatty acids may be beneficial for reducing obesity – a review. Nutrients.

[88] Di Girolamo FG, Situlin R, Mazzucco S, Valentini R, Toigo G, Biolo G (2014). Omega-3 fatty acids and protein metabolism: enhancement of anabolic interventions for sarcopenia. Curr Opin Clin Nutr Metab Care.

[89] McDonald C, Bauer J, Capra S, Coll J (2014). The muscle mass, omega-3, diet, exercise and lifestyle (MODEL) study – a randomised controlled trial for women who have completed breast cancer treatment. BMC Cancer.

[90] Wefel JS, Saleeba AK, Buzdar AU, Meyers CA (2010). Acute and late onset cognitive dysfunction associated with chemotherapy in women with breast cancer. Cancer.

[91] Janelsins MC, Kesler SR, Ahles TA, Morrow GR (2014). Prevalence, mechanisms, and management of cancer-related cognitive impairment. Int Rev Psychiatry.

[92] Yurko-Mauro K, McCarthy D, Rom D, Nelson EB, Ryan AS, Blackwell A (2010). Beneficial effects of docosahexaenoic acid on cognition in age-related cognitive decline. Alzheimers Dement.

[93] Mazereeuw G, Lanctôt KL, Chau SA, Swardfager W, Herrmann N (2012). Effects of ω-3 fatty acids on cognitive performance: a meta-analysis. Neurobiol Aging.

[94] Ye S, Tan L, Ma J, Shi Q, Li J (2010). Polyunsaturated docosahexaenoic acid suppresses oxidative stress induced endothelial cell calcium influx by altering lipid composition in membrane caveolar rafts. Prostaglandins Leukot Essent Fatty Acids.

[95] Chalon S (2006). Omega-3 fatty acids and monoamine neurotransmission. Prostaglandins Leukot Essent Fatty Acids.

